# Advocacy training tool for pediatric residents to strengthen firearm safety laws

**DOI:** 10.3389/fped.2022.1095120

**Published:** 2023-01-10

**Authors:** Scott Risney, Hannah Hollon, James Dodington

**Affiliations:** ^1^ Department of Pediatrics, Monroe Carell Jr. Children’s Hospital at Vanderbilt, Nashville, TN, United States; ^2^ Department of Pediatrics, Virginia Commonwealth University, Richmond, VA, United States; ^3^ Department of Pediatrics, Yale University School of Medicine, New Haven, CT, United States

**Keywords:** advocacy, policy, firearms, pediatric trainee, injury prevenition, pediatrics

## Abstract

Firearm injury is the leading cause of death in children and adolescents in the US, surpassing motor vehicle crashes. There is a need for greater legislative advocacy around firearm injury prevention, specifically around safer storage of firearms. A national medical trainee-based program convened in 2021 with the goal of increasing advocacy efforts around common causes of pediatric injury. A focus was to create a set of advocacy training tools that could be utilized by a wide variety of stakeholders. The subgroup sought to design policy-based training tools; one focused on general firearm injury prevention principles and another specifically focused on Child Access Prevention (CAP) laws. We explicate the utility of these documents and the need for greater advocacy around pediatric firearm injuries

## Introduction - advocacy surrounding firearm injury and death: A state of the union

Firearm-related deaths in the United States have eclipsed motor vehicle crashes as the most common cause of child and adolescent mortality (1). Simultaneously, mass casualty events involving firearms in schools and at public gatherings have catapulted firearm injury prevention into the forefront of pediatric advocacy, successfully galvanizing support from the lay-public as well. In addition to the tragedies captured in the news, every day in the United States children die from firearm-related homicides, suicides, and accidental injuries (2). Children as young as two are capable of pulling the trigger of most handguns, creating dangerous and potentially tragic circumstances for pediatric patients and their families (3). This epidemic of firearm-related injury coupled with increased access to firearms in the homes of children across the United States underlines the need for policy and education initiatives that ensure the safety of children both in and out of the home (4, 5).

Pediatricians are seen as clinical leaders but also advocates for children. In 2016–2017, a widely publicized legal case focused on a law restricting healthcare providers’ ability to ask families about firearm ownership and storage in Florida, and Pediatricians were central to overturning this law, and advocating for firearm safety. Despite this, introductory materials available for pediatric trainees who wish to get started in advocacy are few and far between. Since 2013, the Accreditation Council for Graduate Medical Education (ACGME) in the United States has required that all pediatric residencies include training in advocacy-based initiatives. Many training institutions have trainee-led advocacy initiatives, but these are not standardized and can vary from place to place. Lichtenstein et al. also note that there are a variety of methods implemented by residency programs to teach advocacy with subjective measures of evaluation (6). This variability highlights the need to provide trainees with consistent, reliable tools to build their advocacy toolkit.

The overarching goal of this project was to develop an advocacy tool that could be utilized on a local, state, and national scale, as part of a year-long advocacy training program called Trainees for Child Injury Prevention (7). Specifically, we focused on advocating for safer storage of firearms given the firearm injury epidemic described. We sought to design two, one-page documents, with one focused on general principles to strengthen firearm injury prevention laws and another specifically focused on Child Access Prevention (CAP) laws. With this policy template, the intention was for users to take the information provided and mold it into individualized tools to impact greater change in the communities they serve.

## Methods and policy options/implications

In 2021, medical students, residents, fellows, and attending physicians who served as facilitators from a wide variety of academic institutions across the United States joined to work on a program called Trainees for Child Injury Prevention (T4CIP), based at the Center for Injury Research and Policy and Nationwide Children’s Hospital (7). This was a year-long training program, comprised of monthly meetings on a virtual platform. T4CIP brings together a group of pediatrics-focused trainees from across the US, passionate about child injury prevention. Trainees reach out to their peers and mentors at training programs and institutions to ask them to participate in each “Day of Action” and share advocacy information during conferences and on social media. Program sessions last 1–2 h, and faculty meet separately for training, support, and planning. Sessions are led by national leaders in a specific area of injury prevention corresponding to the “Day of Action,” as well as various sessions on topics like legislative advocacy and social media use for healthcare professionals. T4CIP is also sponsored by the American Academy of Pediatrics Section on Pediatric Trainees and the Council on Injury, Violence, and Poison Prevention. Similar efforts to organize pediatric trainees have been supported by groups like the AAP Section of Pediatric Trainees, however, the structured and fully faculty-supported training and implementation on injury prevention advocacy, at the core of this program, is unique in the field (7). Our 2021 program focused on high-powered magnet ingestion prevention and firearm injury prevention efforts throughout the year.

To focus on areas of interest for trainees, T4CIP created multiple subgroups. As members and faculty of the policy subgroup (SR, HH, JD), we wanted to create materials that were easily digestible for those with and without experience in policy spheres, providing a tool for education on pediatric firearm injury prevention policy. The policy subgroup worked to generate two legislative “Guides” which included information from local and national policy initiatives. Once the information was synthesized, the messaging subgroup designed graphics and formatting to organize the content. Content was reviewed by T4CIP faculty and topic experts assembled through collaboration with the American Academy of Pediatrics Council on Injury, Violence and Poisoning Prevention (COIVPP).

The policy “Guides” were shared with participants and community partners in an electronic format. This open-source approach was desired by T4CIP membership to spread information to anyone interested, allowing them to utilize content however they needed (8). This information was also shared on multiple social media platforms on the Day of Action, including a national Twitter chat. Measures of interactions on social media platforms and other mixed messaging were accounted for by T4CIP leadership to hone best-practices.

We encouraged trainees to disseminate these “Policy Guides” to their institutions and community partners. Stakeholders for T4CIP initiatives included organizations at the local level, such as hospital systems, local/national news organizations, and national groups focused on pediatric injury prevention and research.

**Figure 1:**
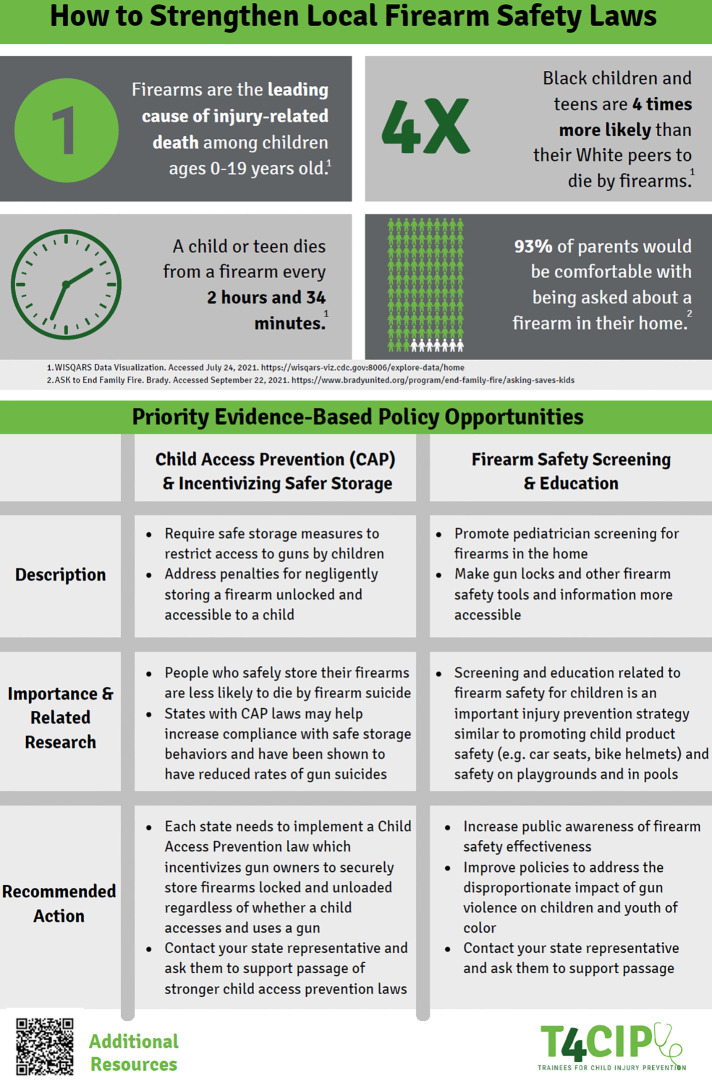
First policy “Guide”: local/regional advocacy part one.

**Figure 2:**
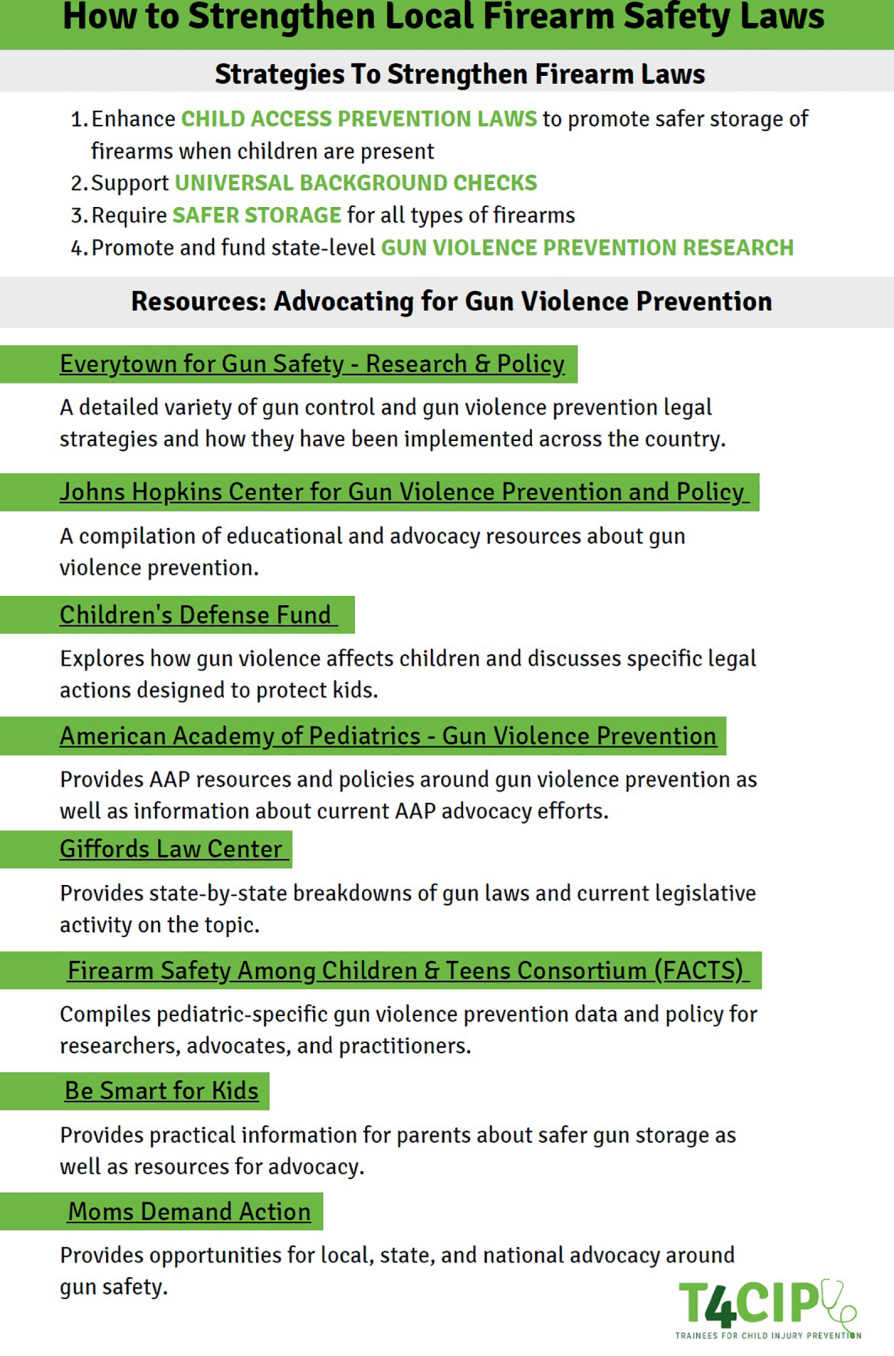
First policy “Guide”: local/regional advocacy part two.

The first policy “Guide” (Figures 1, 2) provided a wide array of information to be utilized at a local/regional level: basic firearm injury statistics to paint the scope of the problem for legislators, policy opportunities to improve safer storage of firearms such as CAP laws and funding for firearm injury education and research, and links to more in-depth resources if needed. Our intention was to develop a framework for stakeholders who presented at differing levels of advocacy familiarity a list of terms that could bring everyone onto the same plane of knowledge. These individuals could then bring this knowledge to local legislators to enact change in their community.

**Figure 3:**
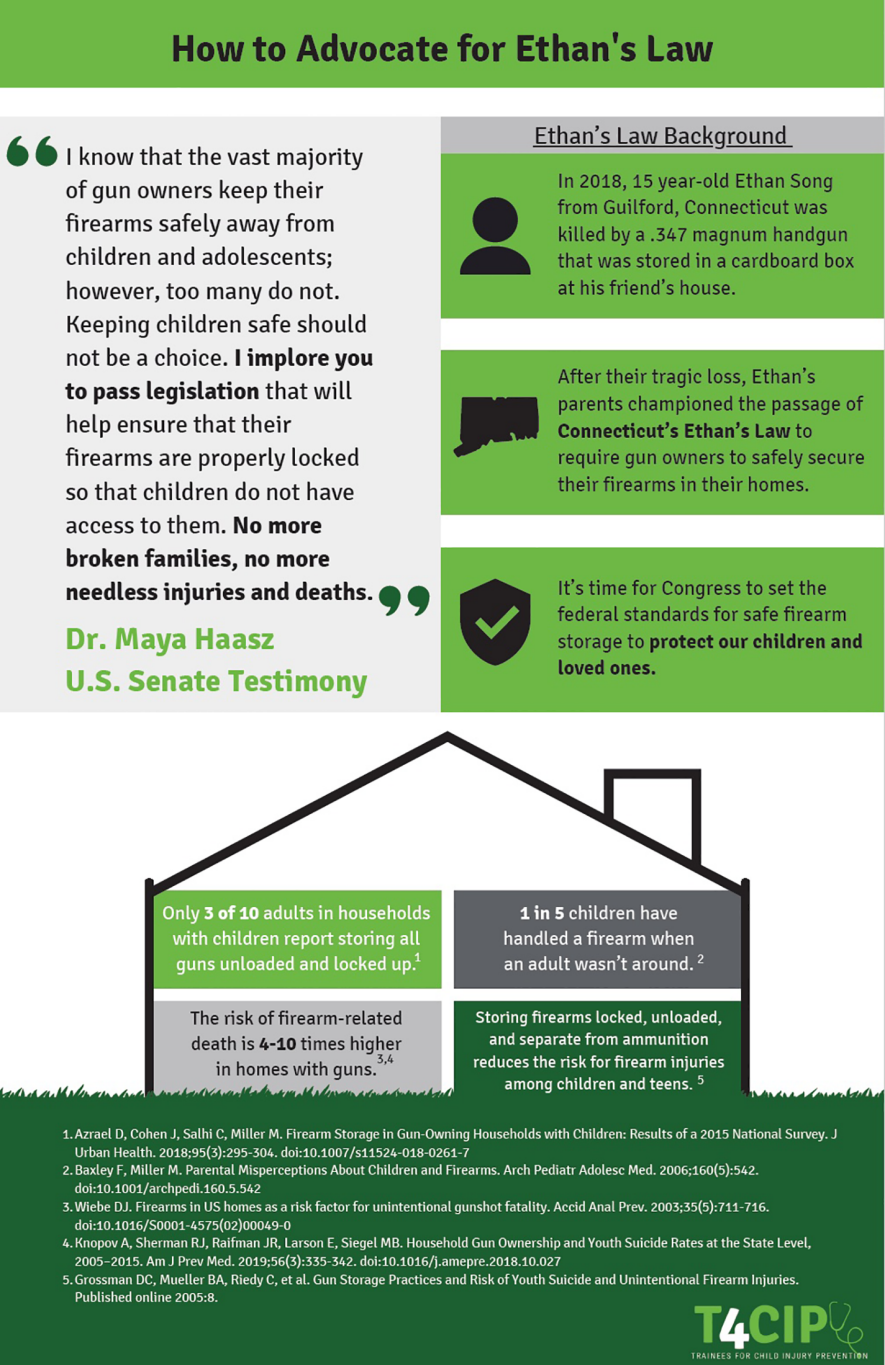
Second Policy “Guide”: Child access prevention laws part 1.

**Figure 4:**
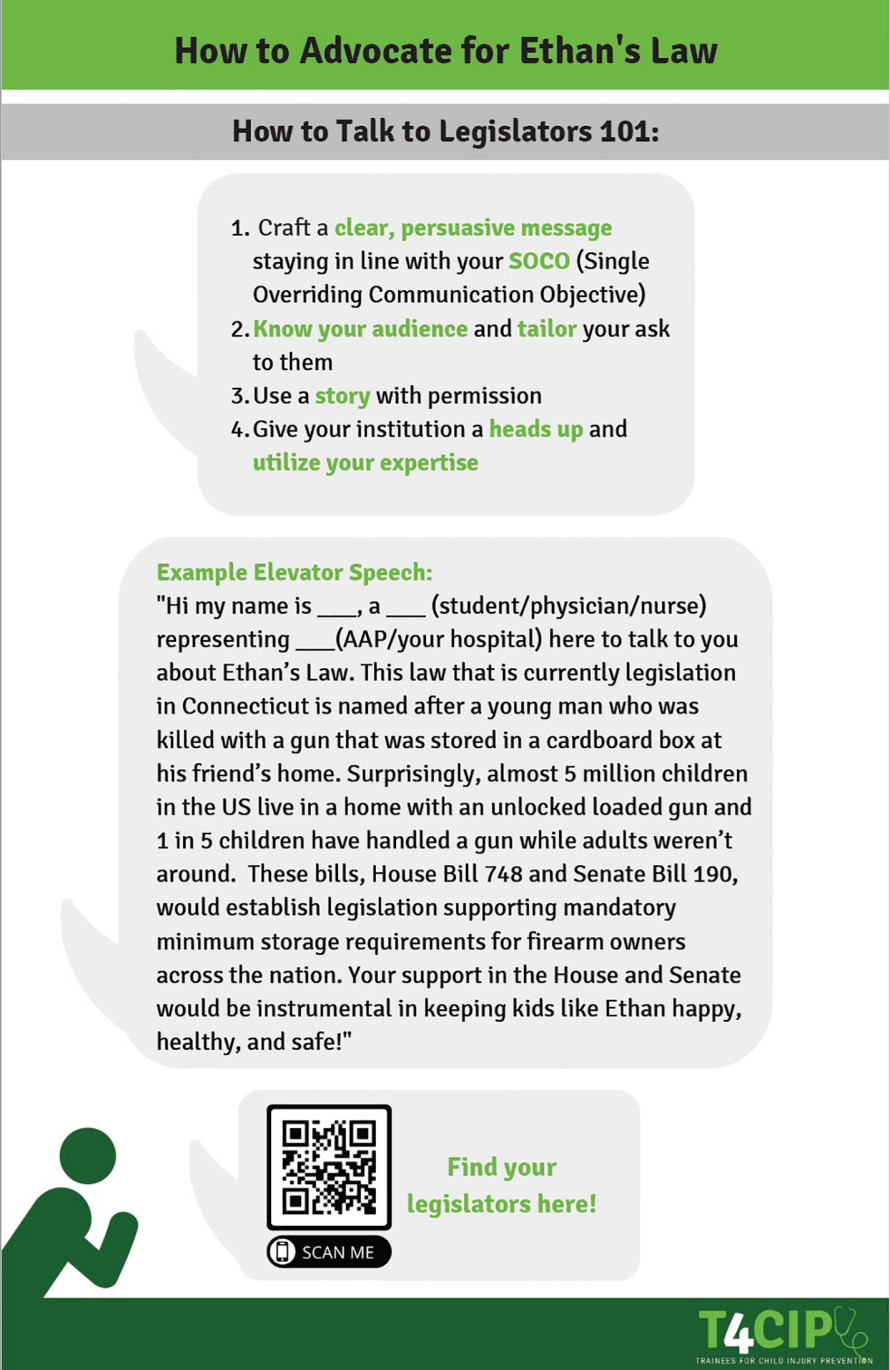
Second Policy “Guide”: Child access prevention laws part 2.

The second policy “Guide” (Figures 3, 4) focused on CAP laws. The state initiative we used as an example was Ethan’s Law, a CAP law passed in Connecticut in 2019 as House Bill 7218 (9). This eponymous law is named after a young man who was fatally killed by a firearm stored at his friend’s house (10). This type of legislation is known as a CAP law, specifically designed to discourage the unsafe storage of firearms in locations where children are present (11). Due to its successful implementation into state law and presentation of similar laws on a national stage, Ethan’s Law served as a perfect example of one that could be brought to local, state, and national attention. There is a federal version of Ethan’s Law that was introduced in 2021 by US Representative Rosa Delauro (D-CT) and US Senators Richard Blumenthal, (D-CT) and Christopher Murphy, (D-CT).

Of note, Child Access Prevention laws such as Ethan’s Law have been implemented in 29 different states and the District of Columbia (11). To date (2022), no federal law has been passed in support of Child Access Prevention for firearms, relying on statewide implementation of laws that vary across borders. This creates an avenue for further change: a push towards creation of laws that keep children safe from firearms in their vicinity at a federal level and across state borders.

We did not seek to provide a comprehensive scientific examination of firearm injuries. Moreso, we sought to provide the reasoning for why this endeavor was undertaken and provide an overview of the problems represented with statistical data. These data could be used in raw form or in the context of more familiar symbology. For example, we opted to use the phrase “a child or teen dies from a firearm every 2 h and 34 min” to utilize a quantitative measure that is more widely understood.

## Outcomes and lessons learned

### Expanding program reach and impact

We measured the reach of our T4CIP Day of Action around safest storage practices for firearms *via* social media data and found 1,050 unique posts, which generated over 3 million impressions of materials. Central to our Day of Action of firearm safety was a keynote seminar for our participants that included Dr. Judy Schaechter, M.D., MBA, the incoming President of the American Board of Pediatrics (at the time) in a question-and-answer session with Dr. Lois Lee, M.D., MPH, a national leader in firearm injury prevention research, policy and advocacy. During the session, key points of our policy document were raised and promoted to the trainees. With each impression during the social media campaign, came the potential that the individual who viewed the post would interact with the policy one-pagers. This dramatically widened the reach of the policy one-pagers, disseminating basic information about firearm injury prevention advocacy and connecting individuals with resources to learn more.

In a previous T4CIP Day of Action, magnet ingestion was highlighted as a troublesome source of pediatric injury. With efforts from T4CIP members, stakeholders, and federal regulators, two main sources of high-powered magnets were recalled citing safety concerns (12). This highlights the power and utility of advocacy efforts around child safety: when pediatric experts advocate for change, real results are possible. Firearm injury prevention efforts are ongoing and widespread, with the potential to touch the lives of 1 in 5 children in the United States, or roughly one entire school bus worth of children per week (13). This has a staggering reach from a public health perspective. We hope to see similar changes from our Safer Storage Days of Action going forward.

### Hone your message and stick to it

Success with regards to the policy briefs was neither static nor binary. One of the most important charges of the policy subgroup was to generate guides that could be used throughout the country and at different times and modified. The documents created provide a framework that members and stakeholders were encouraged to use in their own advocacy efforts. As with many advocacy efforts, there is unlikely to be one defining moment in the success of the initiative. The hope stemming from T4CIP is that members and stakeholders implement and re-work these documents, furthering the reach and messaging of the original work in a more tailored policy approach.

The hyper-focused political atmosphere this work was created under is neither isolated nor transient. It is an environment that creates strain on advocacy efforts and can lead to walking a tightrope when it comes to generation of materials. Challenges that we faced when drafting these “Guides” included the politicized nature of firearm safety policy. To counteract this, our group chose to narrowly focus the message on safer storage of firearms in order to keep children safe. We found these techniques to be successful.

During the Day of Action around firearm safe storage practices, a social media influencer with a background in more “pro second amendment” content became aware of the T4CIP efforts (14). This was a pivotal moment in the advancement of the policy agenda, as someone with social capital could make or break the momentum of the initiative. Due to the focus of T4CIP around childhood safety, this particular individual connected positively with messaging and amplified it to thousands of their followers. As mentioned by Betz et al., “words matter” when it comes to discussing firearms, so choosing language that was both patient-centered and non-stigmatizing to victims and firearm-owners alike was very important to us (15). Readers will notice that we incorporated terminology recommendations from that seminal paper in this publication. This provides an important lesson as one of the keys to success in the advocacy realm: have a message and stick to it.

Lessons learned from this process included maintaining a clear and simplified message. We found that this was a message that many can get behind. We also found it helpful to refer to legislation already in effect at the state level, such as Ethan’s Law, as a model or template for other states to follow. Our messaging team worked closely with firearm owners to design graphics that accurately represented firearms, ammunition, and locks/safes as well as graphics that were inclusive and included people of all backgrounds as firearm injury is not unique to just one group. Due to firearms’ wide footprint across the United States, we also found it helpful to develop materials that could be useful in all fifty states.

### Actionable items

For the policy opportunities, we also wanted to give the description, evidenced-based support for the importance of the recommendation, and the specific actionable items for legislators. In addition to these more specific goals, we provided overarching objectives of firearm injury prevention at large to frame the specific interventions. Two policy options were Child Access Prevention Laws and firearm safety screening and education. In order to foster advocacy-minded pediatricians, training programs should consider educational sessions on the basics behind effective advocacy initiatives for their trainees. Utilizing documents such as an advocacy toolkit, trainees can increase their understanding of how advocacy can have a significant impact in the lives of their patients.

### Child access prevention (CAP) laws and firearm safety

CAP laws are fundamental pieces of legislation that require safe storage measures to restrict access to firearms by children. These laws codify penalties for those individuals who would store a firearm without a locking device and have it used to harm a child. This negligent storage of the firearm could be reinforced if a child used a firearm on themselves or another individual. Research has shown that individuals who safely store their firearms are less likely to die by suicide, and states who have implemented CAP laws have seen reduced rates of self-injury by firearms (16, 17). Our recommendation is to promote the adoption of state-specific and national CAP laws to incentivize owners of firearms to store them locked and fully unloaded. We also sought to create a narrative around the story behind Ethan’s Law, utilizing quotations from a recent United States. Senate testimony by Dr. Maya Haasz, MD with the hope that legislators connected strongly with the story.

In addition to specific examples of why CAP laws are important, to help facilitate conversations with legislators we provided a sample “elevator pitch” that trainees could use to start the conversation about CAP laws. Our hope was to surmount the barrier of knowing what to say to legislators during conversations surrounding CAP laws, with the hope that individuals could utilize the script and broaden it as they saw fit. We also provided a general framework for how to speak to legislators effectively to allow individuals to see where this potential conversation fit into an effective “elevator pitch” for a bill.

Firearm Safety and Screening necessitates that those giving recommendations be well-versed in the specifics of best practices surrounding firearm safety. Screening and educational opportunities provided to pediatric trainees have been shown to be an important injury prevention strategy in other arenas such as child product safety (car seats, bike helmets, ect.) and safety on playgrounds and in pools. When viewed broadly, firearms are just another potentially dangerous product pediatricians and pediatric trainees should have the resources to effectively counsel their patients and caregivers on. Our aim is to increase public awareness surrounding the effectiveness of firearm safety education when delivered by trusted healthcare providers who are informed on the subject.

## Conclusions

As a model, T4CIP was started with the goal to educate, empower, and engage future advocates in pediatric healthcare. Future classes of T4CIP trainees will engage in advocacy efforts focused on high need areas of child injury prevention. Feedback from the original class was obtained and will be incorporated into the curriculum for classes beyond. Allowing trainees space to “learn-by-doing” in order to create similar policy “Guides” around the future Days of Action will allow participants and stakeholders to benefit from the rich collaborative atmosphere that T4CIP has developed.

The hope continues to be that stakeholders and future members would be able to use the briefs in their entirety or as a framework for whatever policy sphere they find themselves in. Future T4CIP classes will be charged with generating similar documentation around the efforts they choose to advocate for, amassing a foundation of policy documents that will help pediatric practitioners in their quest for greater childhood health.
